# Tolerance induced via TLR2 and TLR4 in human dendritic cells: role of IRAK-1

**DOI:** 10.1186/1471-2172-9-69

**Published:** 2008-11-24

**Authors:** Valerie Albrecht, Thomas PJ Hofer, Brian Foxwell, Marion Frankenberger, Loems Ziegler-Heitbrock

**Affiliations:** 1 Helmholtz Center München, German Research Center for Environmental Health and Asklepios-Fachkliniken Gauting, Clinical Cooperation Group, Inflammatory Lung Diseases, 82131 Gauting, Germany; 2 Imperial College, The Kennedy Institute for Rheumatology, London, UK

## Abstract

**Background:**

While dendritic cells (DCs) can induce tolerance in T cells, little is known about tolerance induction in DCs themselves. We have analysed tolerance induced in human *in-vitro *generated DCs by repeated stimulation with ligands for TLR4 and TLR2.

**Results:**

DCs stimulated with the TLR4 ligand LPS did show a rapid and pronounced expression of TNF mRNA and protein. When DCs were pre-cultured for 2 days with 5 ng LPS/ml then the subsequent response to stimulation with a high dose of LPS (500 ng/ml) was strongly reduced for both TNF mRNA and protein. At the promoter level there was a reduced transactivation by the -1173 bp TNF promoter and by a construct with a tetrameric NF-κB motif. Within the signalling cascade leading to NF-κB activation we found an ablation of the IRAK-1 adaptor protein in LPS-tolerant DCs. Pre-culture of DCs with the TLR2 ligand Pam3Cys also led to tolerance with respect to TNF gene expression and IRAK-1 protein was ablated in such tolerant cells as well, while IRAK-4 protein levels were unchanged.

**Conclusion:**

These data show that TLR-ligands can render DCs tolerant with respect to TNF gene expression by a mechanism that likely involves blockade of signal transduction at the level of IRAK-1.

## Background

Tolerance of immune cells will occur when leukocytes down-regulate their response after a primary encounter with antigen or other ligands. Tolerance can act via deletion, receptor down-regulation, blockade of signal transduction or via the action of suppressive cytokines. Monocytes and dendritic cells (DCs) belong to the innate immune system but they are instrumental in instructing the cells of the adaptive immune system via antigen presentation and cytokine production. One of the main cytokines produced by these cells is tumor necrosis factor (TNF), a cytokine that acts at various levels in order to promote immune response and inflammation.

TNF is produced upon activation of monocytes and DCs by microbial products like lipopolysaccharide (LPS) of Pam_3_Cys, which act by binding to toll like receptors (TLRs). When cells are exposed to these compounds repeatedly, then the TNF production is decreased, i.e. the cells have become tolerant. The molecular mechanism of tolerance to date has been studied only in monocytes and macrophages. For LPS stimulation it was shown early on that the CD14 co-receptor was not down but rather up-regulated in tolerant cells [1]. Also, signal transduction still occurred with mobilisation of p50/p65 of NF-κB, but there was at the same time an increase in NF-κB p50-homodimers which bind to the promoter and displace the p50/p65 heterodimer. Since p50 cannot transactivate this will lead to blockade of TNF gene expression [1,2]. In addition to this p50-homodimer mechanism, blockade in LPS tolerant monocytes can also occur through interruption of the signalling cascade at the level of IRAK-1 in that this adaptor protein is proteolytically degraded [3,4]. For monocytes tolerant to the TLR-ligand Pam_3_Cys this mechanism also applied and there was a strong and complete ablation of IRAK-1 [5].

Different mechanisms of tolerance operate in different cell types in that in lymphocytes deletion of cells is a major mechanism, while in monocytes blockade of signal transduction predominates. Because of this tissue specificity in tolerance mechanisms we have induced and analysed tolerance in mature human DCs.

When looking at tolerance induction in DCs Karp et al. [6] and demonstrated that pre-culture with a low dose of LPS led to a 80% reduction of IL-12 protein production upon secondary stimulation with a high dose of LPS. Other than that little is known about tolerance in mature DCs after TLR-ligation and the molecular mechanisms have not been studied. We show herein that tolerance can be induced in dendritic cells both with TLR2 and TLR4 ligands and that the mechanism involved is the ablation of the adaptor molecule IRAK-1. Such tolerance of DCs, which is characterised by lack of proper production of TNF and IL-12, will contribute to reduced immune responses in clinical settings like sepsis.

## Results

### Generation of DCs and induction of TNF

**Figure 1:**
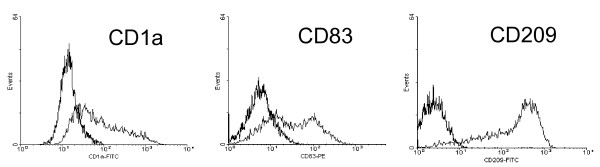
**Cell surface phenotype of monocyte-derived dendritic cells.** Adherent mononuclear cells were cultured for 7 days with GM-CSF and IL-13 with addition of PGE_2 _for the final 2 days. Cells were then stained with specific monoclonal antibodies or the respective isotype controls and analysed in flow cytometry. Percentage of positive cells for CD1a were 48.4 ± 20.7%, for CD83 39.9 ± 14.5% and for CD209 89.5 ± 8.1%. Shown is one representative of 9 experiments.

We generated DCs by culturing of PBMC for 5 days with GM-CSF and IL-13 followed by further culturing for 2 days with addition of PGE_2_. The cell surface phenotype of DCs was determined by flow cytometry and as shown in Fig. 1 the cells generated in this way exhibit a high level expression of CD1a, CD209 and CD83. In average of 9 experiments there were 48.4+/- 20.7% positive cells for CD1a, 39.9 +/- 14.5% positive cells for CD83 and 89.5 +/- 8.1% positive cells for CD209. These data demonstrate that the cells generated are mature DCs.

**Figure 2:**
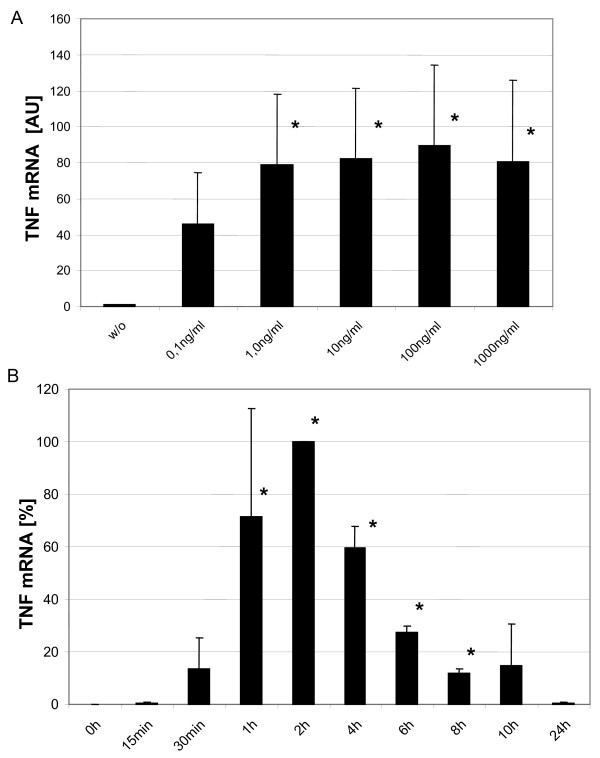
**Dose response and time course analysis for LPS induced TNF mRNA in monocyte-derived DCs. *A*.** Day 7 dendritic cells were stimulated with different doses of LPS from *Salmonella abortus equi *and after 2 hours TNF mRNA levels were determined by RT-PCR. All mRNA levels were adjusted to α- Enolase as house keeping gene. TNF mRNA expression was then calculated in AU while setting the value for TNF levels in unstimulated cells as one. Given is mean of 3 experiments. * = p < 0.05 compared to w/o. *B*. Dendritic cells were stimulated for different times with 500 ng/ml of LPS from Salmonella abortus equi and TNF mRNA levels were determined by RT-PCR. The maximum TNF mRNA expression level at 2 hours was set as 100%. Adjustment to α- Enolase was done as mentioned for Fig. 2A. Given is mean of 3 experiments. * = p < 0.05 compared to 0 h.

We then studied expression of the TNF gene in these day 7 DCs after stimulation with highly purified LPS from *Salmonella abortus equi*. We can show in dose response analysis a 40-fold induction already at a LPS dose of 0.1 ng/ml with an 80-fold induction at 1 – 1000 ng/ml (Fig. 2A).

Time course analysis revealed an early peak at 2 hours post addition of LPS with only 10% of the maximum level left at 8 hours and a return to the unstimulated level at 24 hours (Fig. 2B).

### Induction of LPS tolerance in DCs

**Figure 3:**
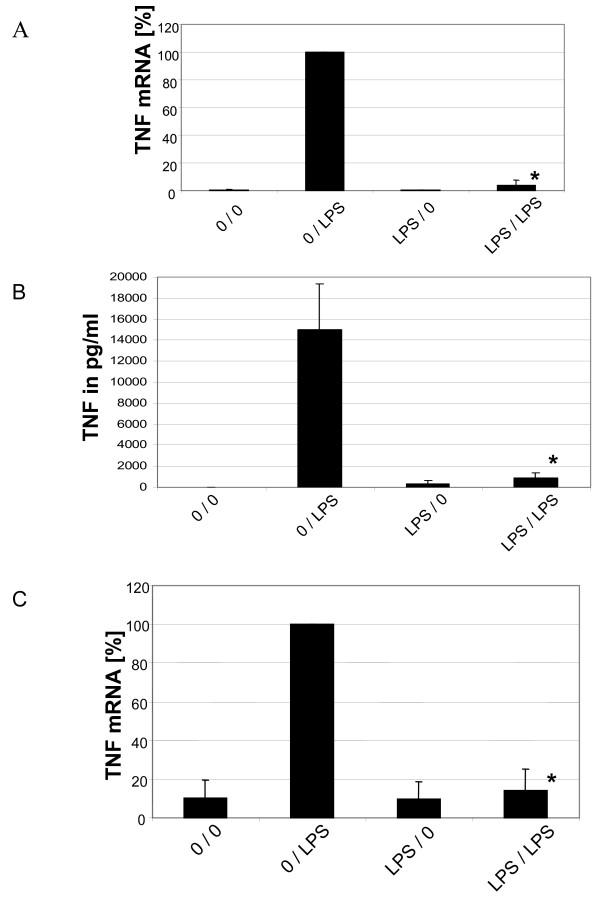
**Induction of LPS tolerance in dendritic cells.***A*. Induction of LPS tolerance in dendritic cell cultures at the level of TNF mRNA at day 7 dendritic cells were cultured for additional 2 days with LPS at 5 ng/ml and then washed and stimulated at 500 ng LPS/ml. Cells were lysed at 2 hours and RNA was extracted, transcribed into cDNA and amplified by RT-PCR and data were normalized relative to levels of the house keeping gene alpha-Enolase. Given is the average of 7 experiments. * = p < 0.05 compared to 0/LPS. *B*. Induction of LPS tolerance in dendritic cell cultures at the level of TNF protein. Day 7 dendritic cells were cultured for additional 2 days with LPS at 5 ng/ml and then washed and stimulated at 500 ng LPS/ml for 6 hours. Supernatants were then harvested and were tested for TNF protein by ELISA. Given is the average of 3 experiments. * = p < 0.05 compared to 0/LPS. *C*. Induction of LPS tolerance in CD83^+ ^dendritic cells at the level of TNF mRNA. Day 7 CD83^+ ^dendritic cells were isolated by MACS to > 90% purity and these cells were then cultured for additional 2 days with LPS at 5 ng/ml, washed and stimulated at 500 ng LPS/ml. Cells were lysed and RNA was extracted, transcribed into cDNA and amplified by RT-PCR and data were normalized relative to levels of the house keeping gene alpha-Enolase. Given is the average of 3 experiments. * = p < 0.05 compared to 0/LPS.

We then used these mature day 7 DCs and pre-cultured them for another 2 days without or with LPS at 5 ng/ml. When cells were left untreated during the preculture period and then stimulated with LPS at 500 ng/ml on day 9 then we could observe a 205-fold induction of TNF mRNA (Fig. 3A, average of n = 7). Cells pre-cultured with LPS at 5 ng/ml and stimulated on day 9 at 500 ng/ml responded with a much lower TNF mRNA expression, which was only 8-fold (Fig. 3A).

The same pattern was seen when testing 6 h supernatants for TNF protein. Here naïve cells stimulated with LPS produced an average of 15 000 pg TNF/ml, which is a greater 1000-fold stimulation compared to 9 pg/ml in unstimulated cells (Fig. 3B). Tolerant DCs stimulated with LPS only produced 900 pg TNF/ml reflecting a 3-fold induction compared to unstimulated tolerant cells (Fig. 3B). These data demonstrate an almost complete blockade of TNF gene expression in LPS-tolerant DCs.

Since DCs were generated from blood monocytes isolated by adherence there were still some contaminating lymphocytes remaining at day 7 of culture. In order to demonstrate that DCs were in fact the responder cells in this system we isolated CD83^+ ^cells by MACS separation to greater 90% purity and subjected them to LPS pre-culture followed by LPS stimulation. As shown in Fig. 3C the purified CD83^+ ^DCs gave the same pattern of response with a pronounced induction of TNF mRNA in the naïve cells and no response in cells pre-cultured with LPS.

### Promoter activity and signal transduction in LPS tolerance in DC

**Figure 4:**
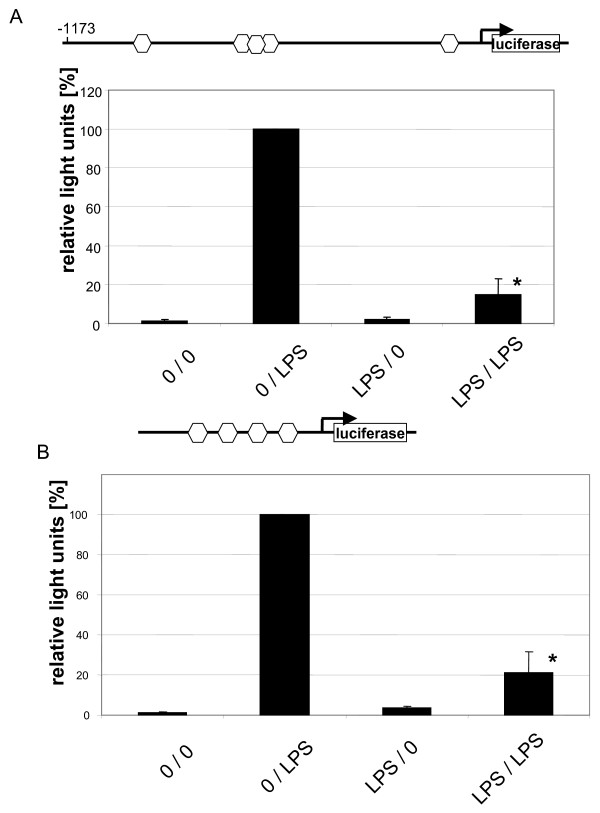
***A*. Reduced TNF promoter activity in LPS tolerant dendritic cells.** Day 7 dendritic cells were pre-cultured without and with LPS at 5 ng/ml and cells were infected on day 8 with the human – 1173 bp TNF promoter luciferase reporter adenovirus. On day 9 cells were stimulated for 5 hours with LPS at 500 ng/ml and luciferase activity was determined and normalized to protein content. Given is the average of 4 experiments. 100% represents 329560 +/- 373169 RLU in these experiments. * = p < 0.05 compared to 0/LPS. The hexagones denote NF-κB binding sites in the human TNF promoter at -873, a complex site from -627 to -589 including a site at – 605, and a site at -104 relative to transcription start. *B*. Reduced NF-κB activity in LPS tolerant dendritic cells. Day 7 dendritic cells were pre-cultured without and with LPS at 5 ng/ml and cells were infected on day 8 with a luciferase reporter adenovirus driven by a tetramer of NF-κB motifs. On day 9 cells were stimulated for 5 hours with LPS at 500 ng/ml and luciferase activity was determined and normalized to protein content. Given is the average of 3 experiments. 100% represents 13803,67 +/- 2651,93 RLU in these experiments. * = p < 0.05 compared to 0/LPS. The hexagones denote NF-κB binding sites.

Expression of the TNF gene is to a large extent governed by its promoter. In order to analyse whether LPS tolerance in DCs operates at the promoter level we infected DCs with an adenovirus containing the human -1173 bp TNF promoter up-stream of the luciferase reportergene. This was done one day into the pre-culture without or with LPS and after culture with the virus overnight cells were stimulated with LPS and luciferase activity was measured. Figure 4A demonstrates a strong induction of the promoter by LPS in naïve cells (in average 83.3 -fold). DCs made tolerant by pre-culture with LPS showed only a 12.0-fold increase in promoter activity (Fig. 4A).

One of the crucial transcription factors that control the TNF gene is NF-κB [7-10]. We therefore asked whether LPS tolerance in DCs is regulated by this transcription factor. For this we infected DCs with an adenovirus containing 4 copies of a NF-κB binding motif upstream of the luciferase reporter gene and analysed the response to tolerance induction. As shown in Fig. 4B the NF-κB dependent luciferase was strongly induced in naïve cells (in average 96.8 -fold) and this was down to only 20.4 -fold in LPS tolerant cells. Taken together, these data show that LPS tolerance in human DCs operates at the level of the TNF promoter and is crucially dependent on NF-κB.

**Figure 5:**
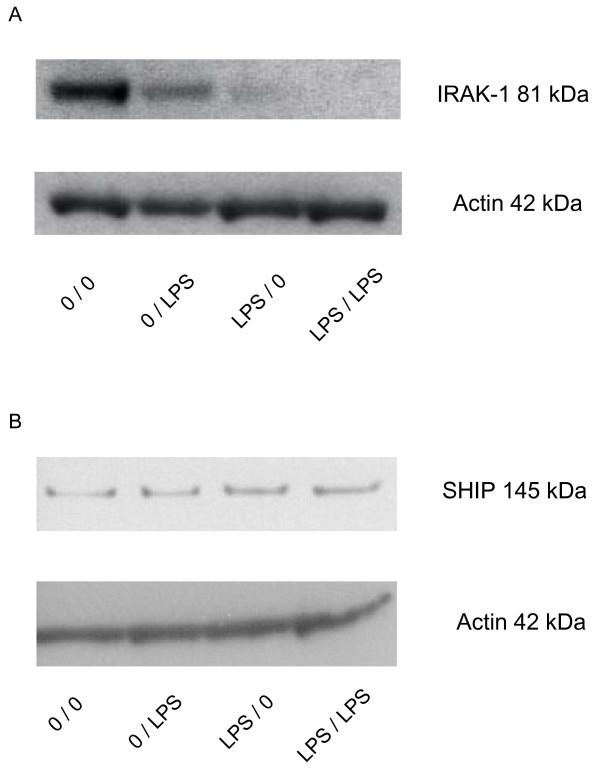
**Effect of LPS tolerance induction on levels of IRAK1 protein.** Day 7 dendritic cells were cultured for additional 2 days with LPS at 5 ng/ml and then washed and stimulated at 500 ng LPS/ml. Lysates were prepared at 1 h and were subjected to Western blotting for IRAK-1 (***A***) or SHIP (***B***). One representative of 4 experiments.

LPS is a prototypic stimulus of monocytes and in these cells it acts via CD14, MD2 and TLR4 cell surface receptors with TLR4 transmitting the signal into the cells by engaging molecules like MyD88 and TRIF. The MyD88 pathway involves recruitment of IRAK-1, which initiates a signalling cascade that leads to activation of IKKs and these will phosphorylate I-κB. I-κB is degraded and allows for NF-κB to be released and to go into the nucleus and transactivate genes like TNF. Blockade of signal transduction has been reported to occur at various levels in LPS tolerance and here the predominant mechanisms are a) up-regulation of p50 homodimers and b) ablation of IRAK-1 [1-4]. In preliminary experiments we could not detect an increase in p50 homodimers in tolerant DC (not shown). Therefore, we have asked whether tolerance to LPS in DCs may be due to blockade at the level of IRAK-1. As shown by Western blotting IRAK-1 is clearly detectable in naïve DCs but after induction of tolerance it is strongly reduced (Fig. 5A). In average of 4 experiments LPS tolerant cells (pre-treated without additional stimulation, lane 3 Fig. 5A) showed IRAK-1 at a level of 20.5 +/- 7.7% of the naïve cells (p < 0.05). Under the same conditions SHIP protein did not show any change with the tolerant cells giving only a slightly higher expression level of the protein (Fig. 5B, 3 versus 1: 157.2% +/- 98.8). These data suggest that LPS tolerance in human DCs acts by interrupting signal transduction at the level of IRAK-1.

### LPS tolerance leads to blockade of IL-12 expression

**Figure 6:**
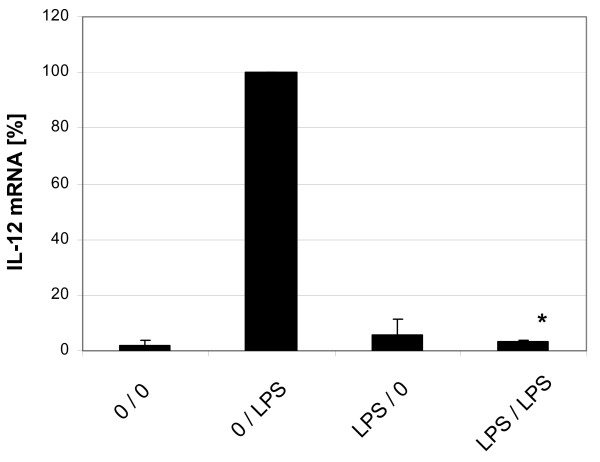
**Induction of LPS tolerance in dendritic cell cultures: effect on IL-12 mRNA.** Day 7 dendritic cells were cultured for additional 2 days with LPS at 5 ng/ml and then washed and stimulated at 500 ng LPS/ml. Cells were lysed at 6 hours and RNA was extracted, transcribed into cDNA and amplified by RT-PCR and data were normalized relative to levels of the house keeping gene alpha-Enolase. Given is the average of 3 experiments. * = p < 0.05 compared to 0/LPS.

IL-12 is another prominent cytokine in DCs and it is also controlled by the transcription factor NF-κB. We therefore have asked whether LPS tolerant DCs will also down-regulate IL-12p40. RT-PCR analysis of lysates taken after 6 hs of stimulation of naïve cells did show a 50-fold induction of IL-12p40 mRNA (Fig. 6). By contrast, DCs pre-cultured with a low dose of LPS followed by stimulation with a high dose of LPS did not show any induction of IL-12 mRNA anymore. Looking at CD80 and CD86 no significantly different expression was seen in tolerant cells. Also, expression of IL-10 was inducible by LPS in naïve dendritic cells and inducibility was maintained in tolerant cells (data not shown).

### Pam_3_Cys Tolerance in DCs

**Figure 7:**
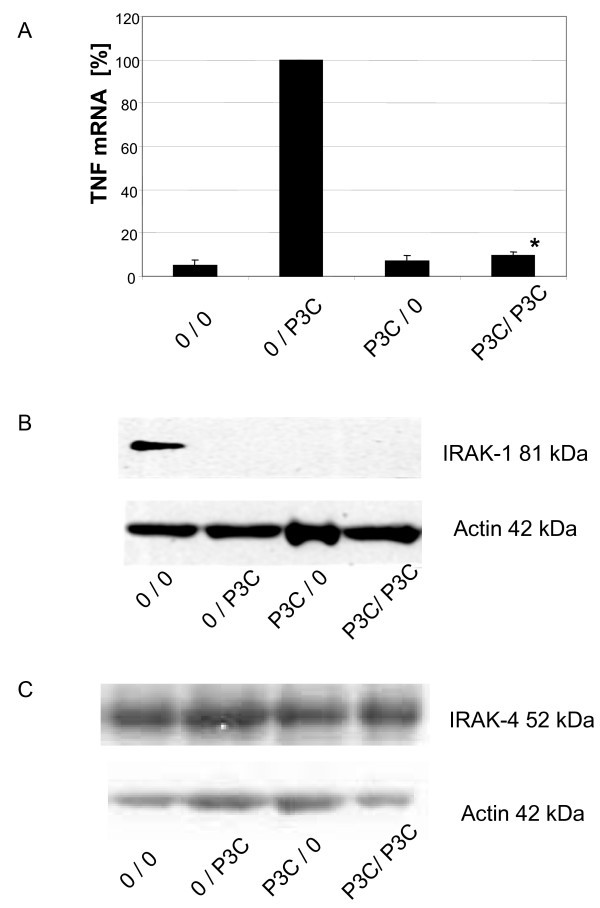
**Induction of Pam_3_Cys tolerance in dendritic cells and effect on levels of IRAK1 protein. **Day 7 dendritic cells were cultured for additional 2 days with Pam_3_Cys at 1μg/ml and then washed and stimulated at 10 μg Pam_3_Cys/ml. *A*. Cells were lysed at 2 hours and RNA was extracted, transcribed into cDNA and amplified by RT-PCR and data were normalized relative to levels of the house keeping gene alpha-Enolase. Given is the average of 5 experiments. * = p < 0.05 compared to 0/LPS. *B*. Lysates were prepared at 1 h and were subjected to Western blotting for IRAK-1. One representative of 3 experiments. *C*. Lysates were prepared at 1 h and were subjected to Western blotting for IRAK-4. One representative of 3 experiments.

Tolerance in monocytes can be induced via LPS targeting TLR4 and via Pam_3_Cys targeting TLR2. We have analysed TNF expression in human DCs pre-treated without or with Pam_3_Cys for 2 days at 1 μg/ml followed by stimulation with Pam_3_Cys at 10 μg/ml. As shown in Figure 7A, tolerance was readily induced as naïve cells showed a 19.6 -fold induction of TNF mRNA while pre-treated cells exhibited only a 1.9-fold induction.

When we probed cellular extracts from such cells for IRAK-1 then we found again a pronounced depletion of IRAK-1 protein in Pam_3_Cys pre-treated cells (Fig. 7B). Here Pam_3_Cys stimulation for 1 h led already to an almost complete depletion of IRAK-1 protein and this continued to be low in Pam_3_Cys tolerant cells on day 2 (lane 3). In average of 3 experiments the IRAK-1 protein level in tolerant cells on day 2 was 1.2 +/- 0.3% as determined by densitometry. At the same time IRAK-4 protein levels remained unchanged (Fig. 7C).

## Discussion

Tolerance is a principle mechanism in biology and describes the absence of a response after repeated stimulation of a cell. Tolerance in the immune system can operate via deletion of reactive cells, receptor downregulation, blockade of signalling and suppression via cytokines. TLR-ligand induced tolerance in monocytes/macrophages has been studied extensively and blockade of signal transduction appears to be the main mechanism of tolerance in these cells. Here blockade has been demonstrated to operate at various levels along the signalling cascade and this includes failure of MyD88 to be recruited to TLR4 [11], up-regulation of IRAK-M [12] or SHIP [13], up-regulation of suppressor of cytokine signalling-1 [14,15] and down-regulation of MAP-kinases [16].

A major mechanism of tolerance that can be demonstrated in macrophages but also in T cells and tumor cells is the up-regulation of p50 mRNA and increased translocation of p50-homodimers into the nucleus [1,17,2-22]. More recently the proteolytic degradation of IRAK-1 has been shown to represent another major mechanism [4,3,23-25]. In a study on cells that were generated over 5 days in the presence of IL-4, GM-CSF and LPS, a reduced TNF production was noted when compared to cells generated with IL-4 and GM-CSF only [26]. The cells generated with LPS did, however, fail to express the DCs marker CD83 and it appears that TNF production was compared between 2 different types of DCs rather then in mature DCs that were pre-cultured without and with LPS. For mature dendritic cells TLR-mediated tolerance has been addressed only in 2 reports [6,27] and for human DCs a down-regulation of IL-12 protein was demonstrated when cells, pre-cultured with a low dose of LPS, were stimulated with a high dose of LPS [6]. The molecular mechanism of tolerance in these cells has, however, not been studied.

We have analysed tolerance to TLR-ligands in the human system and for this have generated monocyte-derived DCs via 7 day culture with GM-CSF, IL-13 and PGE_2_. PGE_2 _was used in the final 2 days in order to induce DCs to a mature stage. We have used this step deliberately since maturation with standard signals like LPS or TNF may induce tolerance on their own. The cells generated in our study were clearly DCs based on their expression of CD1a, CD83 and DC-SIGN. Stimulation of such cells was done with LPS from *S. abortus equi*, which was highly purified LPS to remove any contaminant lipoprotein such that TLR4 but not TLR2 was targeted. This LPS induced a rapid production of TNF mRNA which was still high at 1 ng/ml with a decreased response at 0.1 ng/ml. For induction of tolerance to LPS we used a low dose of LPS (5 ng/ml) and after pre-culture for 2 days stimulated with a high dose of 500 ng/ml, i.e. a 100-fold higher dose. The low dose still was capable to almost completely block the TNF production by the high dose of LPS, thus demonstrating the efficiency of blockade. There was, however, some residual TNF expression in the tolerant cells and this has also been seen in previous studies with tolerant monocytes/macrophages [4,18,1]. This blockade of TNF production in the present study was clearly due to mechanisms operating in DCs since it could be equally demonstrated in purified CD83^+ ^cells (Fig. 3A and 3C).

When addressing the molecular mechanism of this LPS tolerance we initially looked at the activity of the human TNF promoter and of NF-κB restricted reporter constructs. Since DCs are difficult to transfect with standard procedures, we have used adenoviral promoter luciferase reporter constructs for this purpose. These studies demonstrated a reduced TNF promoter activity in tolerant cells and this is similar to what has been shown in LPS tolerant monocytes [1,2]. This suggested that the blockade is at or up-stream of the transcription level. The finding of downregulation of a construct carrying 4-NF-κB motifs furthermore suggested that the NF-κB signalling pathway might be involved. In preliminary gelshift analysis (data not shown) we found no consistent increase of p50-homodimers but rather a failure of NF-κB p50p65 to be mobilised into the nucleus. We therefore went on to study upstream elements by Western blotting. In these studies we found no change in the expression of the SHIP protein (Fig. 5B) but IRAK-1 protein was strongly depleted in LPS tolerant cells (Fig. 5A). Since IRAK-1 is an essential adaptor protein in the signalling cascade, leading from the TLR receptor structure to mobilisation of NF-κB, this depletion of IRAK-1 explains the tolerance of DCs. When looking at tolerance induction after ligation of TLR-2 we also found a strong down-regulation of TNF mRNA and IRAK-1 was also completely ablated by this treatment. The effect of Pam_3_Cys appeared much stronger in this system since IRAK-1 had already completely disappeared after 1 h of treatment with Pam_3_Cys and in cells treated with Pam_3_Cys for 2 days no IRAK-1 protein was detectable (Fig. 7B). Hence, it appears that for both TLR4 and TLR2 ligands the same mechanism of tolerance operate in human DCs. While these data are highly suggestive of a role of IRAK-1 in tolerance of human DCs, over-expression in tolerant cells of IRAK-1 using for instance adenoviral vectors will be required to confirm this conclusion.

The downregulation of IRAK-1 did occur at the protein level, since RT-PCR analysis did not demonstrate any significant change in IRAK-1 transcript levels (data not shown). An additional mechanism of tolerance is the downregulation of TLR-4 such that signal transduction is blocked at the receptor level as described by Nomura et al [28]. We have addressed this point but were unable to obtain detectable cell surface staining with anti-TLR4 in the in-vitro generated DCs, probably due to a low number of receptors on these cells. Analysis of TLR-4 mRNA demonstrated that levels were unchanged in tolerance (data not shown). Still, it is conceivable that in DCs tolerance in response to different stimuli acts at multiple levels including receptors expression, signal transduction and gene expression.

When using different TLR-ligands cross-tolerance can be observed and this has been shown by several groups [29-33]. The study by Sato has clearly shown that cross-tolerance will occur when signalling pathways converge. Hence, TLR2 and TLR4 ligands induce cross-tolerance with respect to TNF via the shared MyD88 pathway and TLR3 and TLR4 ligands do so with respect to IP-10 via the shared TRIF pathway [33]. With respect to dendritic cells a similar scenario can be expected but further studies are needed to address this point.

IRAK-4 is another crucial kinase and adaptor, which interacts with the TLR receptor complex and mediates activation of IRAK-1 and NF-κB [34]. Defects of IRAK-4 can lead to immunodeficiency with decreased cytokine production leading to increased susceptibility to infection with pyogenic bacteria, like staphylococci and streptococci during childhood [35-37]. We therefore have asked whether in tolerant DCs, which do not activate NF-κB and fail to produce cyokines, there may be an additional decrease in expression of IRAK-4. Our data show, however, that this protein is not altered in tolerant DC (Fig 7C).

In human macrophages induction of LPS tolerance has been shown to lead to down-regulation of MHC Class II molecules with subsequent reduction of activation of T cells [38]. In the present study on DCs we could not demonstrate such a decrease, in that naïve cells showed Class II expression at 91.6 +/- 29.1 channels in flow cytometry and LPS tolerant cells were at 83.8 +/- 25.6 channels. Similarly there was no change in expression of MHC Class I in Pam_3_Cys tolerant cells (data not shown). These data suggest that with respect to MHC expression mechanisms of TLR-mediated tolerance are different between macrophages and DCs. We do not know at this stage, whether the tolerant DCs generated in this study will show a reduced activation of antigen specific T cells. We assume, however, that given the almost complete down-regulation of the crucial cytokines TNF and IL-12 tolerance in DCs will lead to inferior activation of TH1 lymphocytes and lower expression of IFNγ by these cells. Such tolerant cells may preferentially activate TH2 cells and non-polarized T cells as described by Langenkamp et al. [39].

In this study we have focused on the expression of the TNF gene but we could also demonstrate blockade of IL-12 gene expression in tolerant DCs. Since IL-12 is also governed by NF-κB transcription factor [40,41] it is conceivable the molecular mechanism of ablation of IRAK-1 is responsible for the blockade of IL-12, as well.

In the present report we have induced tolerance in mature CD83+ dendritic cells, while others have looked at immature cells treated with LPS or TNF plus IL-1beta [6,39,42]. When looking at immature cells the analysis is complicated by the problem that treatment with a given stimulus will induce two processes, i.e. maturation and tolerance, such that it is difficult to define whether down-regulation of a given gene is due to maturation or to tolerance.

We have induced maturation with PGE_2_, which is used for DC generation in many clinical trials and we have found PGE_2 _induced cells to be highly informative for the study of tolerance. While Kalinski et al have reported that such cells show no IL-12 expression [43] we found a robust expression of IL-12 mRNA. This difference may be explained by the fact that Kalinski et al added PGE_2 _from the start of culture, while we have added this compound only for the final 2 of the 7 days of culture.

For the purpose of this study we have not used maturation with LPS [44], TNF [45,46] or a combination of TNF and IL-1β [42], since maturation with all of these stimuli may induce a state of tolerance, such that we cannot study the mechanisms of tolerance in response to LPS anymore.

In addition to tolerance via TLR-ligands the pro-inflammatory cytokine TNF has also been shown to induce tolerance in monocytic cells and here a mechanism of c/EBP interference with NF-κB phosphorylation was demonstrated [47,48]. The dose of LPS used for pre-culture in the present study (5 ng/ml) will induce TNF and this TNF could contribute to tolerance via the c/EBP mediated interference with NF-κB. Such an additional mechanism may come into play in settings where the depletion of IRAK-1 is incomplete.

While the present work has been done on in-vitro generated human DCs it remains to be shown whether similar mechanisms will operate in primary DCs isolated from tissue. Such tolerance of primary dendritic cells after TLR- ligation in settings of infection will likely contribute to a reduced immune response, which may be detrimental but could also be beneficial in that an over-reaction of the immune system is avoided.

## Conclusion

Our study provides the first data on molecular mechanisms on tolerance in DCs. We show that TLR-ligands can render DCs tolerant with respect to TNF gene expression by a mechanism that likely involves blockade of signal transduction at the level of IRAK-1.

## Methods

### Culture medium

Culture medium was RPMI 1640 plus non-essential amino acids, L-glutamine, Penicillin, Streptomycin, oxalacetate, pyruvate and insulin . This medium was passed through a U2000 column (Gambro Medizin-Technik, Planegg-Martinsried, Germany) in order to remove any inadvertently introduced LPS, and this was followed by addition of 1% (v/v) of a pool of human serum obtained from apparently healthy male donors.

### Cells and stimulation

Heparinised (10 U/ml) venous blood was obtained from healthy volunteers after informed consent. The study was approved by the ethics committee of the Ludwig-Maximilians University Munich with the number 279/4. PBMC were isolated by density gradient separation and were seeded at 5 × 10^6 ^cells/ml in 2 ml volumes of medium in 6 well plates (# 3506 Costar, Bodenheim, Germany). After incubation for 1 h non-adherent cells were removed and fresh 2 ml medium containing GM-CSF (50 ng/ml, Leukine^®^, #1111180, Megapharm, St. Augustin, Germany) and IL-13 (50 ng/ml, generously provided by IDM, Paris, France) was added. After culture at 37°C for 5 days PGE_2 _(0.85 μM, # PG-007, Biomol, Hamburg, Germany) was added for another 2 days.

For induction of tolerance to LPS these day 7 DCs were harvested and seeded at 1 × 10^6^/ml in 2 ml volumes in 24-well ultra low-attachment plates (# 3476, Costar) without or with LPS (5 ng/ml, Salmonella abortus equi, kindly provided by Dr. Chris Galanos, Freiburg, Germany, [49]. The dose of 5 ng/was chosen to be well in the plateau of the titration curve and at the same time in the lower range in order to allow for sensitive detection of any decrease induced by the secondary stimulation. After 2 days cells were stimulated without or with LPS at 500 ng/ml.

For induction of tolerance to Pam_3_Cys day 7 DCs were pre-treated with or without the compound (#L2000, EMC microcollections, Tuebingen, Germany) at 1μg/ml in 24-well ultra low attachment plates (#3476, Costar) at 1 × 10^6 ^cells/ml for two days followed by stimulation with or without Pam_3_Cys at 10 μg/ml.

### Flow cytometry

Cell surface staining of dendritic cells was done by incubation of the cells for 30 min on ice with CD1a-FITC (#555806), CD83-PE (#556855) and CD209-FITC (#551264) or the respective isotype controls (all from BD Pharmingen, Heidelberg, Germany). Also, we used anti-HLA DR- PC5 (PN IM2695, Beckman-Coulter, Krefeld, Germany).

At least 1 × 10^5 ^cells were then analysed on an EPICS-XL flow cytometer (Beckman-Coulter) and signals were expressed as specific mean fluorescence intensity, i.e. mean fluorescence intensity of the CD or anti-MHC antibody minus mean fluorescence intensity of the isotype control.

### Isolation of CD83^+ ^cells

Day 7 dendritic cells were reacted with anti-CD83-Phycoerythrin (#556855, BD Pharmingen) followed by microbead-conjugated anti-PE antibody (#130-048-80, Miltenyi-Biotech, Bergisch-Gladbach, Germany). Cells were then purified using a paramagnetic LS column according to manufacturer's instructions (#*130-042-401, Miltenyi)*.

### RT-PCR

Cells (2 × 10^4^) were lysed in 200 μl TRI-Reagent (#T-9424, Sigma, Deisenhofen, Germany) and incubated for 5 min at RT. To this we added 3 μl tRNA (5,67 μg/μl) plus 40 μl Chloroform and RNA was isolated from the water phase as per manufacturer's instruction. The mRNA was reverse transcribed using MuLV RT (N8080018, Applera, Darmstadt, Germany). The quantitative PCR was performed using the LightCycler^® ^system (Roche Diagnostics, Mannheim, Germany) according to the manufacturer's instructions by using the primer pairs as noted below. In brief, 3 μl of cDNA was used for amplification in the SYBR Green format using the LightCycler – FastStart DNA Master SYBR Green I kit from Roche (#2 239 264, Mannheim, Germany) in a total volume of 20 μl per capillary. The following primer pairs were used: 5' α-Enolase: 5' GTT AGC AAG AAA CTG AAC GTC ACA 3'; 3' α-Enolase: 5' TGA AGG ACT TGT ACA GGT CAG 3'; 5' TNF α: 5' CAG AGG GAA GAG TTC CCC AG 3'; 3' TNF α: 5' CCT TGG TCT GGT AGG AGA CG 3'; 5' IL-12p40: 5' CCA AGA ACT TGC AGC TGA AG 3'; 3' IL-12p40: 5' CTG TGT TGC CTT ATC TGG GT 3'.

Fluorescent signals generated during this informative log-linear phase were used to calculate the relative amount of template DNA. Signals for TNF were normalised against the α-Enolase house keeping gene using the LightCycler^® ^Relative Quantification Software (Version 1.0, Roche).

### Reporter gene analysis

Day 7 dendritic cells were seeded at 1 × 10^5 ^cells in 200 μl MM6-Medium + 1% HS in 96-well low attachment-plates (Costar) and they were either left untreated or treated with LPS at 5 ng/ml. On day 8 cells were infected with adenovirus 10:1 MOI using a TNF promoter luciferase reporter gene virus carrying the -1173 TNF promoter in front of the luciferase reporter gene and downstream the 3' UTR, as given in [9] or a 4 × NF-κB luciferase reporter gene virus [50]. On day 9 cells were stimulated without or with LPS at 500 ng/ml for 5 hs followed by assay of luciferase activity. For this cells were resuspended in 250 μl buffer (#E1501, Luciferase Assay System Promega, Mannheim) and cells were disrupted by 3 freeze thaw cycles. 20 μl aliquots were then analysed in a Luminometer (Sirius, Berthold Detection Systems, Pforzheim, Germany).

### TNF ELISA

For determination of TNF protein DCs were pre-incubated for 2d in the presence or absence of 5 ng/ml LPS. Cultures were then stimulated without or with LPS at 500 ng/ml and supernatants were harvested after 6 h. Cell free supernatants were then tested for protein concentration with commercial ELISA kit systems for TNF (PeliKine-compact™, #M1923, CLB via HISS Diagnostics, Freiburg, Germany) according to the manufacturer's instructions. Sensitivity of the ELISA kit was 1 pg protein/ml.

### Western blotting

Cell lysates were obtained by resuspending the cell pellet in 4 volumes of buffer A (10 mM HEPES pH 7,9, 10 mM KCl, 1,5 mM MgCl_2 _plus 2 mM DTT, Aprotinin at 10 μg/ml, 0,1 M PMSF) and after sonication nuclei were pelleted and the supernatant (cytosol) was obtained and admixed with an equal volume of 60% glycerol to be stored at -80°C. Protein concentrations were determined using Bradford reagent in the NanoDrop^® ^ND-1000 Spectrophotometer (NanoDrop Technologies, Wilmington, USA) using bovine serum albumin as a standard. Lysates were separated using pre-cast NuPage™ Bis-Tris-Gels (4–12%, Invitrogen) using a NOVEX EI9001-XCELL™ II Mini Cell. Proteins were then blotted onto nitrocellulose-membranes (Hybond™ECL™). Blots were blocked with dry milk powder and then reacted with primary antibodies rabbit anti-human actin, (#A-2066, Sigma-Aldrich), mouse anti-human IRAK-1 (#sc-5287, Santa Cruz Biotechnology), rabbit anti-human IRAK-2 (#905-264, Biomol), rabbit anti-human IRAK-4 (#905-266, Biomol) or mouse anti-human SHIP1 (#sc-8425, Santa Cruz Biotechnology). This was followed by incubation with the appropriate peroxidase-conjugated secondary reagent (anti-mouse IgG, #A-7282 Sigma-Aldrich or anti-rabbit IgG, A-0545 Sigma-Aldrich). Membranes were then reacted with the ECL™ Western Blotting Detection Reagent (#RPN2106, Amersham Biosciences, Freiburg) and exposed to Hyperfilm™ECL (#RPN3103, 18 × 24 cm, Amersham). Numeric values were obtained by densitometry analysis of the exposed films. Values were corrected with reference to the actin level and data were expressed as % of the signal obtained in untreated cells, which was set at 100%.

### Statistical analysis

Statistical analysis was performed using Students' T-test.

## Abbreviations

DC: dendritic cell; TLR: toll like receptor; LPS: lipopolysaccharide; IRAK: interleukin-1 receptor associated kinases; TNF: tumour necrosis factor.

## Authors' contributions

VA performed most of the experimental work. TH also contributed to the experiments. BF provided reagents and was involved in the planning of the project. MF supervised the project and contributed to the planning and design of the project and performed some experiments. LZ-H designed the project and wrote most of the manuscript.
